# Communication of mechanically ventilated patients in intensive care units

**DOI:** 10.5935/0103-507X.20160027

**Published:** 2016

**Authors:** Carina Isabel Ferreira Martinho, Inês Tello Rato Milheiras Rodrigues

**Affiliations:** 1 Escola Superior de Saúde do Alcoitão - Alcabideche, Portugal.; 2 Center of Interdisciplinary Investigation in Health, Institute of Health Sciences, Universidade Católica Portuguesa - Lisbon, Portugal.

**Keywords:** Communication, Nonverbal communication, Communication barriers, Speech-language pathology, Mechanical ventilation, Intensive care units

## Abstract

**Objective:**

The aim of this study was to translate and culturally and linguistically adapt the Ease of Communication Scale and to assess the level of communication difficulties for patients undergoing mechanical ventilation with orotracheal intubation, relating these difficulties to clinical and sociodemographic variables.

**Methods:**

This study had three stages: (1) cultural and linguistic adaptation of the Ease of Communication Scale; (2) preliminary assessment of its psychometric properties; and (3) observational, descriptive-correlational and cross-sectional study, conducted from March to August 2015, based on the Ease of Communication Scale - after extubation answers and clinical and sociodemographic variables of 31 adult patients who were extubated, clinically stable and admitted to five Portuguese intensive care units.

**Results:**

Expert analysis showed high agreement on content (100%) and relevance (75%). The pretest scores showed a high acceptability regarding the completion of the instrument and its usefulness. The Ease of Communication Scale showed excellent internal consistency (0.951 Cronbach's alpha). The factor analysis explained approximately 81% of the total variance with two scale components. On average, the patients considered the communication experiences during intubation to be "quite hard" (2.99). No significant correlation was observed between the communication difficulties reported and the studied sociodemographic and clinical variables, except for the clinical variable "number of hours after extubation" (p < 0.05).

**Conclusion:**

This study translated and adapted the first assessment instrument of communication difficulties for mechanically ventilated patients in intensive care units into European Portuguese. The preliminary scale validation suggested high reliability. Patients undergoing mechanical ventilation reported that communication during intubation was "quite hard", and these communication difficulties apparently existed regardless of the presence of other clinical and/or sociodemographic variables.

## Abstract (pt)

**Objetivo:**

Traduzir e adaptar cultural e linguisticamente o instrumento *Ease of Communication Scale* e determinar o nível de dificuldades de comunicação dos doentes submetidos à ventilação mecânica com entubação orotraqueal, relacionando-o a variáveis clínicas e sociodemográficas.

**Métodos:**

Este estudo teve três fases: (1) adaptação cultural e linguística da *Ease of Communication Scale*; (2) avaliação preliminar de suas propriedades psicométricas; e (3) pesquisa observacional, descritivo-correlacional e transversal, realizada entre março e agosto de 2015, com base nas respostas à *Ease of Communication Scale -* após a extubação, de 31 doentes adultos, extubados e clinicamente estáveis, admitidos em cinco unidades de cuidados intensivos portuguesas, e em suas variáveis clínicas e sociodemográficas.

**Resultados:**

A análise dos peritos revelou elevada concordância em relação ao conteúdo (100%) e à pertinência (75%). O pré-teste obteve elevada aceitabilidade ao nível do preenchimento e da sua utilidade. A *Ease of Communication Scale* apresentou excelente consistência interna (alfa de Cronbach de 0,951). A análise fatorial explicou cerca de 81% da variância total com duas componentes da escala. Em média, os doentes consideraram as experiências de comunicação, durante a entubação, "muito difíceis" (2,99). Não existiu relação estatisticamente significativa entre as dificuldades de comunicação reportadas e as variáveis sociodemográficas e clínicas estudadas, com exceção da variável clínica "número de horas após a extubação" (p < 0,05).

**Conclusão:**

Realizou-se a tradução e a adaptação para o português europeu do primeiro instrumento de avaliação das dificuldades de comunicação dos doentes mecanicamente ventilados nas unidades de cuidados intensivos. A validação preliminar da escala sugeriu elevada fiabilidade. Os doentes submetidos à ventilação mecânica consideraram que as experiências de comunicação durante a entubação foram "muito difíceis" e estas dificuldades de comunicação pareceram existir independentemente da presença de outras variáveis clínicas e/ou sociodemográficas.

## INTRODUCTION

Mechanical ventilation with orotracheal intubation prevents patients from communication by speak. Thus, this situation presumably increases patient vulnerability during hospitalization in an intensive care unit (ICU).^(1,2)^ In recent years, guidelines were established^(3-6)^ indicating that mechanical ventilation should be performed under low levels of sedation, whenever the clinical condition of the patient allows, to reduce the occurrence of other complications, including delirium and/or cognitive and emotional impairment of patients.^(3,6-8)^

Furthermore, communication difficulties often preclude patients from expressing their opinions and, therefore, medical treatment decisions could be made without their knowledge.^(9,10)^ Waking up intubated and ventilated at an ICU was described by some patients as frightening, and the inability to communicate effectively made them feel "trapped in a dysfunctional body" because they could understand everything they were told, yet no communication aid that could enable them to respond effectively was available whatsoever, according to their reports.^(11)^ Those communication difficulties are also experienced by the relatives of mechanically ventilated patients who feel helpless and frustrated because they are unable to understand what their relative wants to communicate. Those feelings are much worse when the patient dies without having the opportunity to communicate verbally.^(12,13)^ Healthcare professionals also report feeling uncomfortable when trying to communicate with patients undergoing orotracheal intubation,^(14)^ thereby limiting their communication with patients to brief interactions regarding clinical procedures.^(15)^ Furthermore, communication difficulties experienced by mechanically ventilated patients are reportedly associated with increased negative emotions and frustration levels.^(14,16-19)^

Recently, the Royal College of Speech & Language Therapists^(20)^ published a paper with guidelines based on the latest evidence; their paper reported that speech therapists/speech-language pathologists should be part of the human resources available at an ICU, performing functions in the areas of swallowing and communication disorders. A recent study^(21)^ assessed the percentage of mechanically ventilated patients at an ICU who benefited from augmentative communication systems and consultancy with a speech therapy/speech-language pathologist and reported that approximately 53.9% of the sample (N = 1,440) met the optimal conditions for such benefits.

The Ease of Communication Scale (ECS) is used in several studies to measure the communication difficulties of intubated patients and may be applied during intubation or after extubation.^(15,18,22-24)^

Communication difficulties experienced by mechanically ventilated patients are a current problem and may be mitigated using communication support programs developed by multidisciplinary teams.^(25-28)^

In Portugal, studies on this subject remain scarce. Furthermore, the assessment of communication difficulties for those patients still lacks a specific instrument for this context that has been translated and linguistically adapted to the Portuguese population.

The present study aimed to: (1) translate and culturally and linguistically adapt the ECS assessment instrument;^(18)^ (2) preliminarily extract the psychometric properties of the ECS - after extubation;^(18)^ and (3) analyze the level of communication difficulties experienced by patients who were mechanically ventilated with orotracheal intubation at Portuguese intensive care units and relate these difficulties to sociodemographic (gender, age and education level) and clinical (sedation levels, number of hours of intubation, number of hours after extubation and cause for intubation) variables.

## METHODS

The present study consisted of three stages. The first stage was dedicated to the cultural and linguistic adaptation (translation and back-translation) of the ECS, the subsequent validation of its content by a panel of experts, the design of the pretest and the final review of the instrument. The second stage included the preliminary evaluation of the psychometric properties of the ECS - after extubation. The third and final stage was dedicated to the observational, descriptive-correlational, cross-sectional study, considering the level of communication difficulties experienced by the participants (according to the ECS - after extubation) as the dependent variable and the sedation level, number of hours of intubation, number of hours after extubation, reason for intubation, age, education level and gender of the participants as the independent variables.

The process of translation and cultural and linguistic adaptation of the ECS was conducted according to the recommended theoretical assumptions,^(29)^ which include the completion of five different steps, namely: translation of the instrument, obtaining a consensus version of both translations, back-translation of the consensus version, review by a panel of experts, and application of the instrument in the pretest.

The ECS pretest was performed with a group of three patients. The scale was applied to each patient during mechanical ventilation with orotracheal intubation and after extubation.

Data were collected from March to August 2015 in five Portuguese polyvalent ICUs at the *Centro Hospitalar Barreiro-Montijo*, EPE; the *Hospital de Vila Franca de Xira*; the *Hospital do Espírito Santo de Évora*, EPE; the *Unidade Local de Saúde de Castelo Branco*, - EPE, and the Hospital Beatriz Ângelo, after approval by the five hospital administration and ethics committees for health.

A convenient non-probabilistic sample was used, and the following inclusion criteria were considered: over 18 years old; having undergone mechanical ventilation with orotracheal intubation, with sedation level 1 or 2, according to the Ramsay Sedation Scale, for at least 6 hours; being extubated, conscious and oriented; being clinically stable; and having signed the informed consent form. The following exclusion criteria were considered: clinical history of psychiatric and/or neurological disease; severe sensory changes (including blindness or severe deafness); illiteracy; inability to speak Portuguese fluently; and period of extubation longer than 72 hours.

The study sample consisted of 31 patients who were admitted to the ICU, primarily males (64.5%; n = 20). Two participants who showed memory difficulties at the time of data collection were excluded from the study, despite meeting the inclusion and exclusion criteria in the first clinical screening. The mean age of the participants was 63.4 years, with ages ranging from 34 to 83 years. Approximately 90% of the participants were Portuguese (n = 28), although two Guinean participants and one Belgian participant, fluent in Portuguese, were also included. The education level ranged from 2 to 16 years, with an average of 6.2 years of literacy.

The ECS - after extubation, a clinical and sociodemographic data form, in which all clinical data were outlined, and the sociodemographic variables gathered from the physician or nurse responsible for the patient were used for data collection. All of the study participants signed an informed consent form.

A rate of agreement among the panel of experts equal to or higher than two-thirds was the basic criterion for the scale validity and content analysis, after its translation and back-translation.^(29)^ The same agreement criterion was also applied to the participants who performed the pretest. The collected data were analyzed using statistical and inferential analyses with the Statistics Package for Social Sciences (SPSS), version 22.0, IBM, 2013.

All of the statistical tests performed considered a 5% statistical significance level (p-value < 0.05).

The only participant whose reason for intubation was associated with "other causes" was excluded from the inferential analysis to avoid compromising the internal and external validity of the inferential conclusions.

## RESULTS

The translation and cultural adaptation of the instrument reached a consensus between the experts regarding its relevance and content. The applicability of the Portuguese version of the ECS was supported by the participants, who performed the pretest and completed the scale, and by the lack of missing data. Regarding the pretest of the scale, the application of the ECS - during intubation had a mean score of 2.7; the total mean score of the test was slightly higher, averaging 2.93 points, when applied to the same patients after extubation.

Data collection among the 31 participants occurred an average of 30 hours after extubation, ranging between two and 69 hours after extubation.

Regarding the clinical variables of the participants, the most prevalent reasons for intubation in the sample were postsurgical complications (n = 17; 54.8%), followed by acute (n = 10; 32.3%) and chronic (n = 3; 9.7%) respiratory diseases. One participant whose intubation occurred because of anaphylactic shock was also included (n = 1; 3.2%).

Approximately 90.4% of the study sample were conscious during the intubation period, with sedation levels of 2 (n = 14; 45.2%) or 1 and 2 (n = 14; 45.2%), according to the Ramsay Sedation Scale. On average, the study participants remained ventilated and intubated with sedation levels 1, 2 or at both levels for approximately 35 hours.

The components analysis was performed after obtaining a value of 0.893 in the Kaiser-Meyer-Olkin test. The Bartlett's sphericity test obtained the value of 279.299 (p < 0.001). The initial factor analysis was performed for all of the individual items of the instrument using the factor extraction method and the scree test, with the result of two components with eigenvalues greater than one that accounted for over 80% of the initial data variance. A principal component analysis (PCA) was used to identify what contributed the most to each component obtained. That analysis confirmed the data obtained in the initial factor analysis, identifying a first component with high weight among all of the variables, except item 8 ("In general, how hard was it for you to communicate your thoughts?"). However, this item had a high weight in the second component.

The ECS - after extubation obtained excellent total internal consistency (Cronbach's alpha = 0.951). The correlation between each item and the total items was considered high (r > 0.7), ranging from 0.77 to 0.91 for all items, except item 8, which had a 0.44 correlation. However, that item did not appear to affect the internal consistency of the instrument because, even if removed, the Cronbach's alpha value remained higher than 0.9.

The mean score of the participants' answers to the ECS - after extubation was 2.99 (0.815 standard deviation and 3.20 median), with a minimum value of 1.5 and a maximum value of 4.

**Figure 1:**
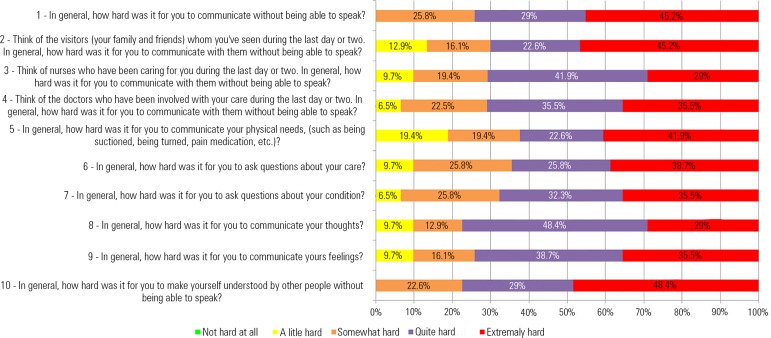
Distribution of the participants' answers to each question in the Ease of Communication Scale - after extubation.

Figure 1 represents the graphical distribution of the participants' answers to each ECS - after extubation question.

A small number of the participants considered the communication experiences during intubation to be "a little hard" or "somewhat hard", in contrast to the high number of participants who considered the communication experiences during intubation to be "quite hard" or "extremely hard". The "a little hard" level was selected in 26 responses, the "somewhat hard" level was chosen in 64 responses, the "quite hard" level was selected in 101 responses, and the "extremely hard" level was selected as a response in 118 times by the study participants.

The joint analyses of the "quite hard" and "extremely hard" levels in the participants' answers showed that 74.2% of the participants reported that "communicating without being able to speak" was "quite hard" or "extremely hard", and 77.4% considered it "quite hard" or "extremely hard" "to be understood without being able to speak". Regarding communication partners, nearly 70% of the participants reported that communicating with physicians (71%) and nurses (70.9%) was "quite hard" or "extremely hard", followed by communication with relatives and friends (67.8%). Communication with relatives had the highest percentage of "extremely hard" answers (45.2%) among the three items, when analyzed separately.

Regarding gender, the mean ECS - after extubation score of the female participants (n = 11) was 2.99 with a 95% confidence interval (95%CI) of 2.54 - 3.41, whereas this value was slightly higher in the male participants (n = 19), with an average score of 3 and a 95% CI of 2.55 - 3.44.

Regarding the reasons for intubation, participants with chronic respiratory diseases (n = 3) had a mean ECS - after extubation score of 3.27, with a 95%CI of 2.64 - 3.89, followed by participants with acute respiratory diseases, with a mean ECS - after extubation score of 3.02, 95%CI 2.53 - 3.51, and individuals who were intubated because of postsurgical complications, with a mean ECS score of 3.01 and a 95%CI of 2.51 - 3.51.

The participants with sedation levels 1 and 2 (n = 14) had a mean ECS - after extubation score of 3.15, with a 95%CI of 2.66 - 3.64, followed by the participants with sedation level 1 (n = 3) with a mean ECS score of 3.10, 95%CI 1.79 - 4.51, and the participants with sedation level 2 (n = 13), with a mean ECS score of 2.90 and a 95%CI of 2.39 - 3.43.

No significant differences were observed between gender (p = 0.611), intubation cause (p = 0.651) and sedation levels (p = 0.635) of the participants and the mean ECS score (Table 1).

**Table 1:** Correlations between mean score and categorical variables

**Categorical variables**	**N**	**Mean score**	**Lower limit**	**Upper limit**	**Mean score**
Gender					
Female	11	2.99	2.54	3.41	0.611*
Male	19	3.00	2.55	3.44	
Reason for intubation					
Postsurgical complications	17	3.01	2.51	3.51	0.651†
Acute respiratory diseases	10	3.02	2.53	3.51	
Chronic respiratory diseases	3	3.27	2.64	3.89	
Sedation level					
1	3	3.10	1.79	4.41	0.635†
2	13	2.90	2.39	3.43	
1 and 2	14	3.15	2.66	3.64	

* Mann-Whitney test;

† Kruskal-Wallis test.

Regarding the numerical variables, a significant correlation occurred between the mean score and the number of hours after extubation. The correlation observed was considered weakly positive (r = 0.360; p = 0.049) and suggested that the increase in the number of hours after extubation led to the increase in the mean score of communication difficulties reported by the patients (Table 2).

**Table 2:** Correlations between mean score of the scale and numerical variables

**Numerical variables**	**Mean score**
Age	
Pearson's r	0.155
p-value	0.421
Number of hours of conscious intubation	
Pearson's r	-0.006
p-value	0.974
Number of hours after extubation	
Pearson's r	0.369*
p-value	0.049
Education level	
Pearson's r	-0.191
p-value	0.32

* The correlation is significant at a 0.05 level (both limits).

## DISCUSSION

The European Portuguese version of the ECS obtained high linguistic and conceptual equivalence when compared to the original version. The instrument was considered relevant, adequate and displayed overall agreement of its content.

Although the primary study objective did not include an analysis of the patients' answers to the ECS, during and after extubation, the pretest results interestingly showed - despite the small sample size - that the patients reported a similar level of communication difficulties in both evaluation times, with a variation of approximately only 0.23 between the mean scale scores. Those data may be key preliminary indicators of the instrument's stability. Moreover, the findings corroborated the results reported by Menzel^(22)^ who found no significant differences between the patients' answers during intubation and after extubation.

The preliminary validation of the ECS - after extubation showed excellent internal consistency, and the reliability obtained was similar to that of previous studies.^(18,23,24)^ Factor and scale component analyses were performed and identified two components responsible for the total data variance.

The mean score of the participants' answers to the ECS - after extubation was 2.99, which shows that the participants generally considered the communication experiences while intubated to be "quite hard". Those mean scores, albeit slightly higher, are similar to those found by Khalaila et al.,^(23)^ Menzel^(18)^ and Liu et al.;^(24)^ the participants of those studies reported moderate levels of communication difficulties. The higher level of communication difficulties of the present study may be related to its smaller sample compared to that in the aforementioned studies.

The results from the participants' answers to each question of the scale demonstrated that more than 74% of the participants considered both the experience of "communicate without being able to speak" and "to make yourself understood without being able to speak" as "quite hard" (29%) or "extremely hard" (45.2% and 48.4%, respectively). The separate analysis showed that no participant considered either item (questions 1 and 10) "a little hard", in contrast with more than 45% participants who selected the answer "extremely hard" to both questions. Those results corroborate some qualitative studies in which the participants considered the experience of communicating under ventilation and the failure inherent to those attempts a very difficult, disturbing and frustrating situation that causes feelings of insecurity.^(10,16,17,19,30)^ Communicating and succeeding in doing so are key factors for patients admitted to the ICU who, in addition to pathophysiological care, also require effective communication tailored to their individual conditions. Accordingly, those difficulties must be the target of specialized interventions for decreasing their negative impact.

The analysis of communication partners showed that the participants reported experiencing more communication difficulties (joint percentage of the answers "quite hard" and "extremely hard") with physicians and nurses than with relatives and friends. Those data corroborate the study by Engström et al.^(30,31)^ wherein the participants reported that communicating with relatives was easier than communicating with the ICU staff. However, the analysis of the answers separately showed that communicating with relatives and friends was most often considered "extremely hard" by the participants (45.2%). Although apparently paradoxal, those results may be related to a higher number of topics that patients want to discuss with their relatives and the complexity of those topics; moreover, meeting with relatives or friends may cause increased emotional susceptibility in a tremendously uncertain situation and, therefore, adversely affect communicative interactions. Communication with nurses received the lowest number of "extremely hard" answers (29%) compared to the other two communication partners, possibly because they are the staff most available for communicative interactions with patients, as reported in a study^(15)^ that found that communication with patients was often initiated by nurses (86.2%). Nevertheless, approximately 41.9% of the participants considered communication with nurses to be "quite hard" in the present study; other studies^(15)^ reported that 40% of the sample cited difficulties in the interaction with nurses, particularly when the communication aimed pain expression (37.7%). Notably, this percentage is also similar to that found in the present study, wherein the communication of physical needs (suction, change of position and pain) was considered an "extremely hard" task by 38.7% of the participants.

The analysis of the sociodemographic variables of the participants showed that communication difficulties occurred regardless of the individuals' gender and age, which corroborates the findings reported by Menzel.^(22)^ The analysis of the participants' level of education in the present study showed no significant relationship between this variable and the level of communication difficulties experienced. Those results contrast with those of the study by Liu et al.,^(24)^ wherein the participants with lower education levels showed more communication difficulties. In the present study, those differences may be explained by the admission of patients with a smaller variation in education level, which tended to be low compared to the education level of the participants included in the study by Liu et al.,^(24)^ wherein 33 of the 80 participants had completed secondary or higher education.

The analysis of the patients' clinical variables showed that neither the number of hours during which the patients remained intubated nor the reason for intubation had any effect on the level of communication difficulties they experienced, similar to the findings by Menzel.^(22)^ However, the results from the present study indicate an effect of the number of hours after extubation on the levels of communication difficulties reported; that is, patients tend to experience increasing communication difficulties as the time after extubation increases. Similar data were reported by Zetterlund et al. in their cross-sectional study,^(32)^ wherein patients' memories of the mechanical ventilation period remained stable, even five years after the first interview. The same authors also reported a significant increase in feelings of anxiety and depression regarding the experience of intubation. These results should be confirmed in studies with a larger sample size. Moreover, the present results indicate the relevance of including participants whose time after extubation is longer than 72 hours; the effect of that variable should be assessed in future studies because the current domestic and international studies tend to include patients within this time period.

Some studies advocate that mechanically ventilated patients with a lower sedation level tend to have more memories of the difficulties level they experienced while intubated.^(3,33)^ That trend could not be confirmed in the present study: there were no significant differences occurring between the sedation level of patients and the difficulties they experienced. Those results may be explained by the sedation levels, which are the lowest of the sedation scale used in the study, and because most of the participants (40.2%) had been under sedation levels 1 and 2 in the last 48 hours of intubation (scale reference time).

The main study limitation was the relatively small sample size. However, considering the specificity of the individuals included, their clinical context, the inclusion and exclusion criteria and the difficulties in accessing the ICU services, the sample included showed a significant representativeness regarding the proposed objectives, achieving encouraging results that corroborate other studies conducted with larger samples.

Although the Portuguese version of the ECS obtained high linguistic and cultural acceptability and the preliminary analysis of its internal consistency showed that the instrument has excellent reliability, further studies should be conducted to improve its accuracy. The reliability should be assessed in future studies through interobserver agreement, and an analysis of its temporal stability should also be performed by applying the test-retest because the results of the scale must be the same when applied by different professionals and at different times. Those assessments were not performed in the present study given the difficulty in involving other ICU staff members and the short period of time during which the patients remained hospitalized in the ICU after extubation.

The adoption of an intervention protocol contemplating the intervention of a speech-language pathologist would also be relevant to an increased awareness of the different methods and modes of communication, considering the communication difficulties for patients in this particular context. Similarly, re-evaluating the communication difficulties for patients using the ECS to assess whether its application had a positive impact on the communication of patients with their respective communication partners after applying the communication protocol in the ICU setting would also be worth investigating.

## CONCLUSION

The Portuguese version of the Ease of Communication Scale showed good psychometric properties and may be a useful instrument in assessing the communication difficulties for mechanically ventilated patients at intensive care units. We believe that the translation and contribution to the validation of the Ease of Communication Scale represent a significant advance in the study of communication difficulties for mechanically ventilated patients at Portuguese intensive care units considering the scarcity of Portuguese studies in this area.

The communication experiences that occurred while patients were mechanically ventilated were considered as "quite hard", tending towards a positive relationship between the perceived level of communication difficulties and the number of hours after extubation. Such difficulties occurred regardless of the existence of other clinical and/or sociodemographic variables.

The difficulties considered "extremely hard" by most of the sample were "to make yourself understood without being able to speak" and "communicate without being able to speak", which were also the only two questions of the scale that no participant classified as "a little hard".

This topic should be the subject of further studies and, where possible, such communication difficulties should benefit from the specialized intervention of a speech therapist. Increased awareness of all healthcare professionals directly dealing with communication difficulties for these patients is also desirable for making healthcare increasingly individualized and targeted and to ensure true autonomy and appreciation for hospitalized patients.
